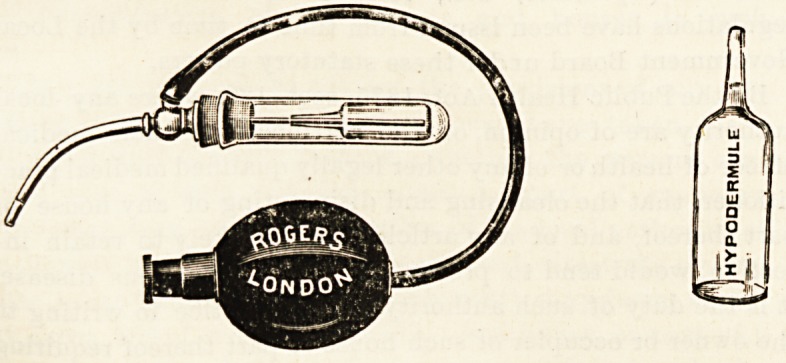# New Appliances and Things Medical

**Published:** 1902-07-19

**Authors:** 


### NEW APPLIANCES AND THINGS MEDICAL.

**[We shall be glad to receive at our Office, 28 & 29 Southampton Street, Strand, London, W.C., from the manufacturers, specimens of all new preparations and appliances which may be brought out from time to time.]**

### NEW MINIATURE NASAL SPRAY AND NASAL HYPODERMULES.

(Frank A. Rogers, 327 Oxford Street, London, W.)

The continued use of Rogers' nasal spray with nasal hypodermules represent the most perfect method yet suggested for the attainment for absolute cleanliness or asepis in nasal treatment. The spray itself involves no new principle; it is small and very neatly made, it fits by means of an indiarubber cork into a small tube, into which the glass capsule containing the medicated solution can easily be slipped. When required for use the neck of the small glass hypodermule is taken off by means of a metal cylinder provided for the purpose. The hypodermule itself is then slipped into the glass tube, and the thin metal delivery tube of the spray is carefully passed down the neck of the hypodermule into the solution. The rubber cork is then fixed in position and the spray is ready for use. By means of the hypodermules it is possible to provide in a perfectly sterile condition such drugs as Adrenalin chloride, which for their therapeutic effect can only be depended upon when preserved by some such method. The solutions supplied by the makers of the spray include those of cocaine, eucaine, heroin, etc. The method appears to us so supremely practical that extended applications, such for instance as those involved in ophthalmic practice, at once suggest themselves as probable in the near future.

### BYNO-CASCARA.

(Allen and Hanburys, Ltd., Bethnal Green, London, E.)

This tonic laxative is one of the most valuable of the malt preparations which has emanated from this most enterprising firm of chemists. It combines the tonic and aperient principles of cascara sagrada and rhamnus Frangula with the digestive properties of extract of malt. It is especially indicated for the use of children, as it is perhaps the most agreeable medicament on the market which contains an adequate dose of cascara in small and tasteful bulk.

## Figures and Tables

**Figure f1:**